# Engineering Hydrogels for Modulation of Dendritic Cell Function

**DOI:** 10.3390/gels9020116

**Published:** 2023-02-01

**Authors:** Cuifang Wu, Lijing Teng, Caiyuan Wang, Tianbao Qian, Zuquan Hu, Zhu Zeng

**Affiliations:** 1 Key Laboratory of Infectious Immune and Antibody Engineering in University of Guizhou Province, Engineering Research Center of Cellular Immunotherapy of Guizhou Province, School of Basic Medical Sciences/School of Biology and Engineering (School of Modern Industry for Health and Medicine), Guizhou Medical University, Guiyang 550025, China; 2 Immune Cells and Antibody Engineering Research Center in University of Guizhou Province, Key Laboratory of Biology and Medical Engineering, Guizhou Medical University, Guiyang 550025, China; 3 State Key Laboratory of Functions and Applications of Medicinal Plants, Guizhou Medical University, Guiyang 550025, China; 4 Key Laboratory of Endemic and Ethnic Diseases, Ministry of Education & Key Laboratory of Medical Molecular Biology of Guizhou Province, Guizhou Medical University, Guiyang 550004, China

**Keywords:** dendritic cells, hydrogels, mechanical cues, immune function

## Abstract

Dendritic cells (DCs), the most potent antigen-presenting cells, are necessary for the effective activation of naïve T cells. DCs encounter numerous microenvironments with different biophysical properties, such as stiffness and viscoelasticity. Considering the emerging importance of mechanical cues for DC function, it is essential to understand the impacts of these cues on DC function in a physiological or pathological context. Engineered hydrogels have gained interest for the exploration of the impacts of biophysical matrix cues on DC functions, owing to their extracellular-matrix-mimetic properties, such as high water content, a sponge-like pore structure, and tunable mechanical properties. In this review, the introduction of gelation mechanisms of hydrogels is first summarized. Then, recent advances in the substantial effects of developing hydrogels on DC function are highlighted, and the potential molecular mechanisms are subsequently discussed. Finally, persisting questions and future perspectives are presented.

## 1. Introduction

As the most potent antigen-presenting cells, dendritic cells (DCs) play important roles in the initiation and regulation of innate and adaptive immune responses [1,2]. For example, tumor-antigen-loaded DCs can activate tumor-specific cytotoxic T lymphocytes, followed by the initiation of anti-tumor immune responses. Unfortunately, clinical trials have shown relatively poor treatment effects [3,4,5]. Therefore, it is crucial to understand how the biophysical and biochemical factors impact DC functions. Our previous studies demonstrated that many suppressive cytokines secreted in the tumor microenvironment impact the motility and immune functions of DCs [6], such as transforming growth factor-β1 (TGF-β1) [7,8], interleukin-10 (IL-10) [9], and vascular endothelial growth factor (VEGF) [10]. Apart from biochemical cues, increasing evidence has demonstrated that biophysical cues also have crucial impacts on DC function, including confinement [11,12,13,14], topology [15], porosity [16], stiffness [11,17,18], and viscoelasticity [19]. As such, engineered hydrogels are emerging as a powerful platform to spatiotemporally manipulate biophysical cues in vitro owing to their extracellular matrix (ECM) mimetic performance, such as high water content, a sponge-like pore structure, and tunable mechanical performance. Additionally, diverse gelation mechanisms have evolved to endow engineered hydrogels with versatile and tunable performance [20,21], such as covalent, noncovalent, and dynamic covalent interactions. Based on these beneficial features, various studies have reported the design of ex vivo models to recapitulate the three-dimensional mechanical microenvironment that DCs sense in vivo. In this review, we aim to highlight the most recent engineering hydrogel developments and how their physical factors affect DC function. The underlying mechanobiology mechanisms are also subsequently discussed.

## 2. Gelation Mechanisms for Producing Hydrogels

**Figure 1:**
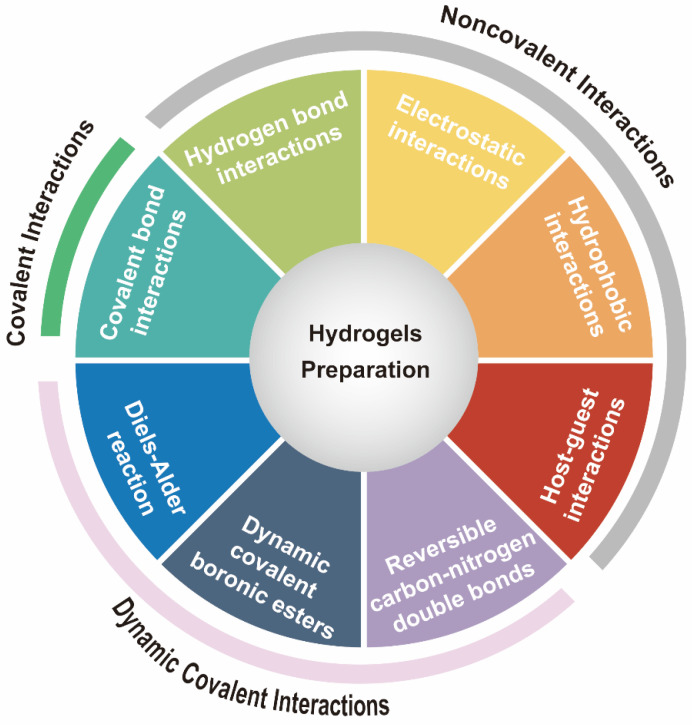
Gelation mechanisms for producing hydrogels.

As shown in Figure 1, many gelation mechanisms have been used for the design and fabrication of various hydrogels, which can be broadly categorized into covalent interactions [22], noncovalent interactions [23], and dynamic covalent interactions [24,25]. In this section, we will briefly review the common gelation mechanisms that can be used as platforms for exploring DC mechano-sensing or migration.

### 2.1. Hydrogels with Covalent Interactions

Covalently crosslinked hydrogels can be developed through chain-growth polymerization [26,27] or step-growth reactions [22]. Chain-growth polymerizations can be initiated with redox initiation or photoinitiation, which subsequently induces the free radical polymerization of reactive groups for rapid hydrogel formation, such as acrylates, meth-acrylates, and acrylamides [28]. Alternatively, step-growth polymerizations enable hydrogel systems in which polymer precursors with different functional groups react by direct mixing [29]. Various hydrogel systems have been developed for two-dimensional (2D) or three-dimensional (3D) cultures of DCs, including polydimethylsiloxane hydrogels (PDMS) [17,30], polyacrylamide (PAM) hydrogels [18], photo-crosslinked polymer hydrogels [31], and thrombin-crosslinked fibrin hydrogels [13].

**Figure 2:**
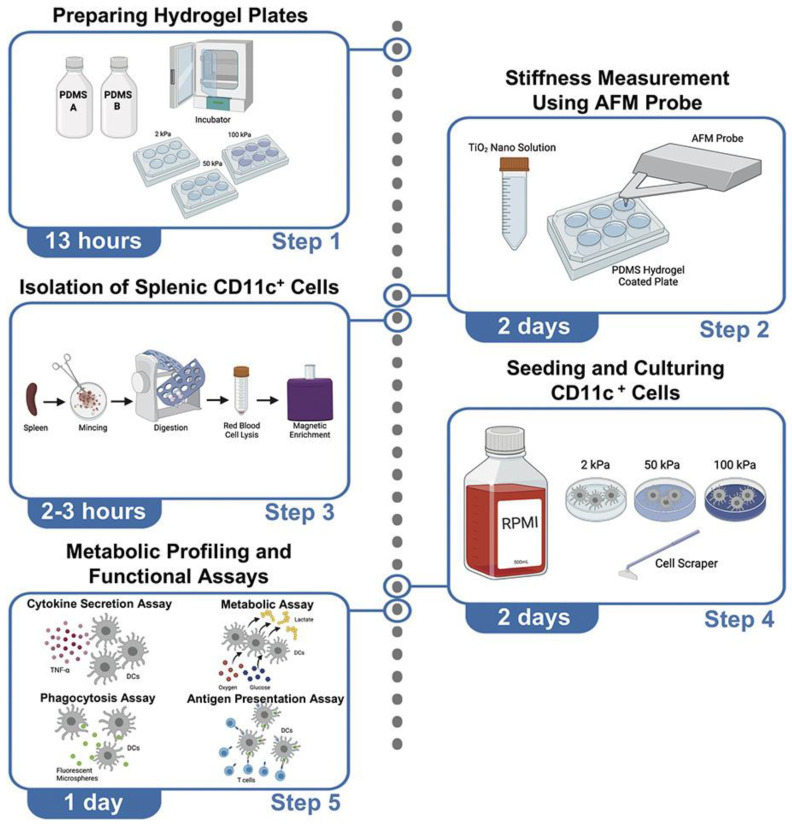
PDMS hydrogel-coated tissue culture plates with different substrate stiffnesses for exploring the impact on DC function (reprinted/adapted with permission from Ref. [30], 2022, Lee et al.).

PDMS-based elastomers are a promising in vitro cell culture platform for studying cellular mechano-sensing owing to their excellent features, such as mechanical elasticity, ease of fabrication, and acceptable biocompatibility [32]. However, the extreme chemical inertness of PDMS limits its applicability. To resolve this issue, various approaches have been proposed for PDMS surface functionalization, such as coating the PDMS surface with a hydrogel [33] or covalently linked proteins [15]. As shown in Figure 2, the PDMS hydrogels were formed using part A and part B components, and the stiffness was increased from 2 to 50 kPa by fixing the ratio of part A part to B components to 1.2:0.3, respectively. Subsequently, the PDMS hydrogels were coated with fibronectin for exploring the impacts of substrate mechanical stiffness on DC differentiation and maturation, metabolism, quality, and function [17,30]. In particular, PDMS can be used as a building block for developing complex environments based on microfluidics, such as micrometric confining channels [15]. Matthieu et al. reported that DCs in complex environments possess a mechanism to pass through micrometric PDMS microchannel constrictions with different restricted sizes, in which the PDMS microchannels were functionalized with fibronectin [15]. Although DC responses to PDMS substrates with patterned microchannels have been extensively studied, the processing parameters for PDMS substrates are usually incompatible with DC encapsulation.

**Figure 3:**
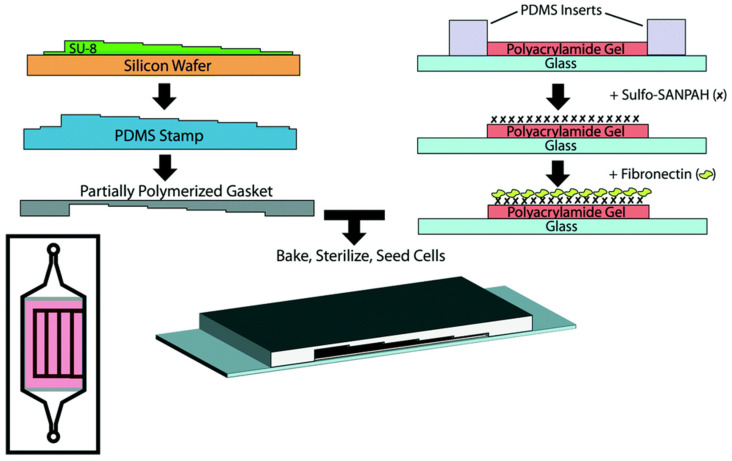
Fibronectin-functionalized PAM hydrogels with varied substrate stiffnesses for cellular responses. Device manufacture and preparation: The PDMS step gasket is developed and then adhered to a coverslip to make a rectangular-shaped PAM hydrogel coated with fibronectin. (Reprinted/adapted with permission from Ref. [37], Galie et al.).

PAM is a polymer (–CH_2_CHCONH_2_–) formed by acrylamide subunits. PAM hydrogels are usually obtained through free radical polymerization of acrylamide and *N,N*-methylene-bis-acrylamide (BIS) bifunctional monomers. PAM hydrogels are a particularly popular platform for cellular mechano-sensing studies owing to their hydrophilic and semipermeable performance [34,35]. Most importantly, the stiffness of PAM hydrogels can be tuned within a wide range, from 0.3 to 300 kPa, by changing the concentration of acrylamide monomer or BIS, which can well provide different in vivo matrix stiffnesses [36]. Nevertheless, PAM hydrogels are biologically inert, lacking cell interaction sites [18]. The surface functionalization of PAM substrates with the ECM has been suggested as a potential strategy to achieve a bioactive surface. Covalently immobilized protein is a particularly popular approach for patterning the ECM on PAM substrates [37,38]. As shown in Figure 3, Janmey et al. reported how the combined substrate stiffness and fluid shear stress impacted endothelial cell behavior. PAM hydrogel substrates with a wide range of stiffness from 100 Pa to 30 kPa were developed by free radical polymerization. After polymerization, the PAM hydrogel substrates were treated with sulfosuccinimidyl 6-(4′-azido-2′-nitrophenylamino)hexanoate (sulfo-SANPAH) and UV-treated, and the hydrogel substrates were then incubated with fibronectin [37]. In 2017, PAM hydrogel substrates with stiffnesses of 2, 12, and 50 kPa were developed by random radical polymerization of acrylamide and BIS monomers, and then human fibronectin was immobilized on the PAM hydrogel substrate for exploring the influences of substrate stiffness on the phenotype and function of DCs [18]. Additionally, most reported PAM stiffness-regulated DC functions were determined in 2D cultures [39], which might not be able to accurately represent the complicated microenvironments of peripheral tissues and lymphoid organs.

**Figure 4:**
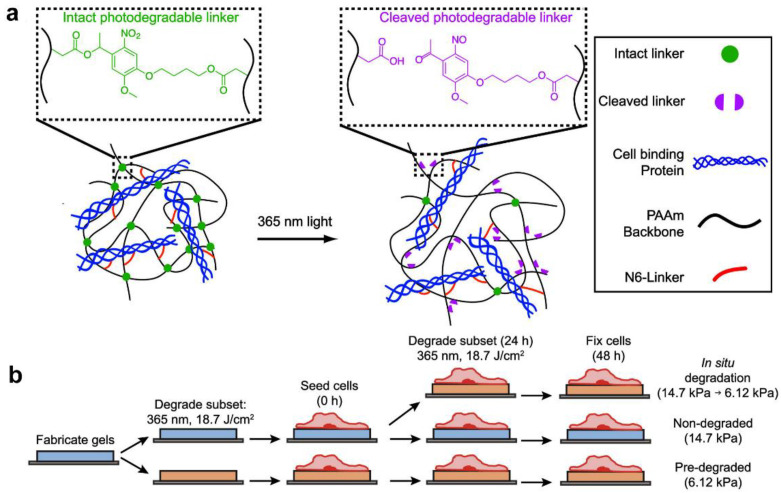
Photodegradable polyacrylamide gels for dynamic control of cell functions. (**a**) Structure of collagen-functionalized photodegradable polyacrylamide hydrogels for dynamic control of cell functions. (**b**) Photodegradable hydrogels were first fabricated and functionalized with an ECM protein. Next, a subset of the gels was softened prior to cell seeding (“pre-degraded”). Then, 24 h after cell seeding, a subset of the non-softened gels cultured with cells were softened in situ (“in situ degradation”), and the remaining gels were left unsoftened (“non-degraded”). (Reprinted/adapted with permission from Ref. [42], 2021, Norris et al.).

Although biophysical studies utilizing PAM hydrogels with fixed stiffnesses have been extensive, cells or tissues constantly remodel their environment and respond to dynamic microenvironments [40]. Incorporating photosensitive linkers into the hydrogel network is one of the more popular methods for simulating this phenomenon [41]. For example, Li et al. developed a photodegradable PAM hydrogel in which the stiffness was dynamically controlled with exposure to ultraviolet light [42]. As shown in Figure 4a, the photodegradable PAM hydrogel was formed by free radical polymerization between acrylamide and o-NB-bis-acrylate linkers, and then collagen type I was conjugated to the photodegradable PAM hydrogel surface by covalent coupling. When the hydrogels were exposed to ultraviolet light, the o-NB crosslinks disconnected, resulting in decreased stiffness. The formed photodegradable PAM hydrogel can be used to explore more complex cellular behaviors (Figure 4b).

**Figure 5:**
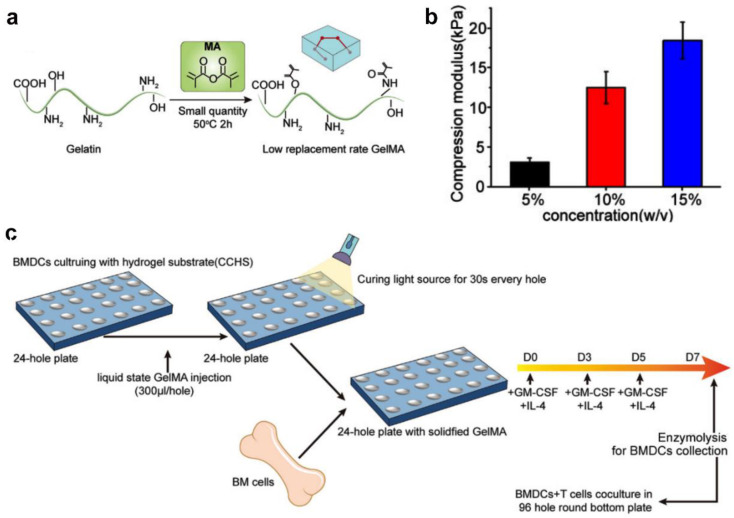
Photocurable hydrogel substrate for BMDC culture. (**a**) Synthetic equation of photo-crosslinked gelatin hydrogel substrate. (**b**) Compression modulus of photo-crosslinked gelatin hydrogel substrate with different concentrations. (**c**) BMDCs were cultured with GM-CSF and IL-4 from C57 mice on photo-crosslinked gelatin hydrogel substrate with different compression modulus. (Reprinted/adapted with permission from Ref. [31], 2022, Deng et al.).

Photo-crosslinking is another well-explored approach for developing covalently crosslinked hydrogels. Photo-crosslinked hydrogels have drawn much attention in in vitro cell culture platforms, as they enable rapid gelation under physiological conditions [26]. The gelatinization can readily occur through exposure of a photosensitive system to ultraviolet or visible light. Additionally, the mechanical performance can be adjusted through simply changing the light intensity, exposure time, concentration of the modified polymer, or degree of graft modification [43]. Many naturally derived biopolymers have been utilized to develop photo-crosslinked hydrogels, such as alginate [20], hyaluronic acid [44], and gelatin [45,46]. Generally, normal bone marrow-derived dendritic cells (BMDCs) cultured on conventional plastic dishes are readily susceptible to activation because the plastic dishes have an elastic modulus that does not actually match the physiological microenvironment in vivo. To explore how to better maintain the BMDC immunophenotype in vitro, Xiang et al. reported a novel in vitro BMDC culturing platform based on a photo-crosslinked gelatin hydrogel substrate that simulated an ECM-like microenvironment and subsequently reduced BMDC activation during culturing (Figure 5a–c). BMDCs cultured in the optimized gelatin hydrogel substrate displayed parallel phenotypes and functions to spleen DCs in vivo and were capable of facilitating T cell stimulation after lipopolysaccharide (LPS) activation [31]. The reported photo-crosslinked gelatin hydrogel substrate has the potential for exploring the immune response mechanism of DCs in vitro and subsequently improving the development of DC vaccines.

In addition to photo-crosslinked naturally derived biopolymer hydrogels, photo-crosslinked synthetic polymer hydrogels also provide cell culture platforms with tunable mechanical performances or distinct patterns for exploring the response of cells to mechanical cues [47]. For example, poly(ethylene glycol) diacrylate (PEGDA) is a hydrophilic and biocompatible polymer that forms a hydrogel network through photopolymerization, in which crosslinking occurs under physiological conditions, and can be used to explore cellular responses to biophysical cues in both two and three dimensions [48]. Additionally, the mechanical performance of PEGDA hydrogels can be regulated by changing the concentration or molecular weight of PEGDA, and an increased elastic modulus can be achieved with a decrease in molecular weight or an increase in polymer concentration [49]. Nevertheless, PEGDA is practically the same as PDMS and PAM hydrogels in that none of these replicate native ECM-like architectures and composition; however, this can be resolved by introducing proteins or polypeptides into the hydrogel network using physical or chemical strategies [50,51].

**Figure 6:**
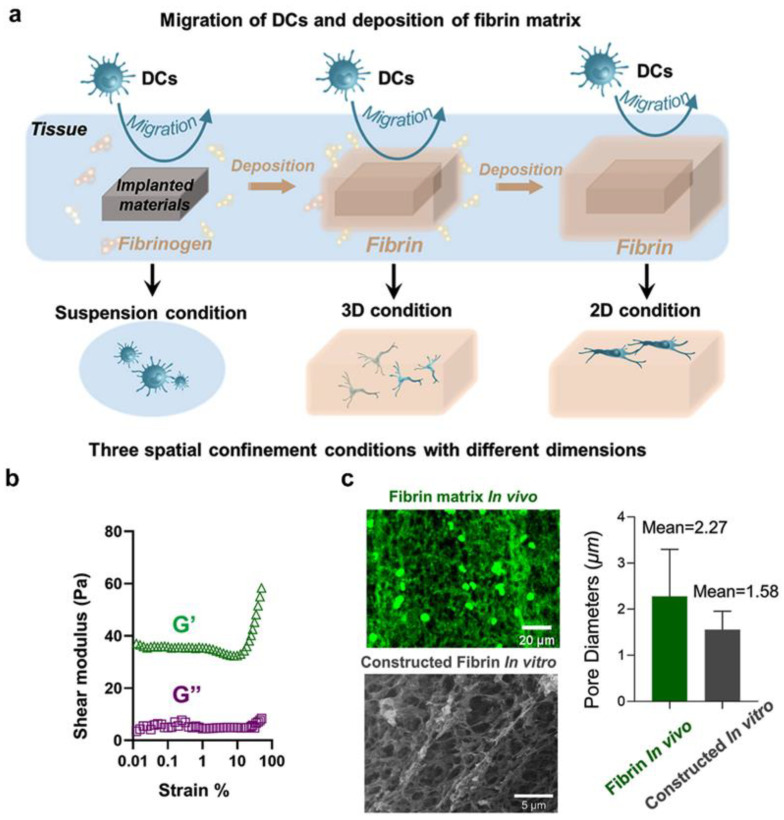
Fibrin hydrogel for DC culture. (**a**) Schematic diagram of fibrin hydrogel for DC culture. (**b**) Rheological performance of fibrin matrix. (**c**) Structure of fibrin matrix in vivo and in vitro, respectively. (Reprinted/adapted with permission from Ref. [13], 2022, Hu et al.).

Fibrin derived from the polymerization of fibrinogen is a viscoelastic biomacromolecule with both elastic and viscous performance, which is attributable to its reversible mechanical deformation and irreversible deformation, respectively [52]. In the formation of a fibrin hydrogel network, fibrinogen transforms itself into fibrin by the action of thrombin [53]. Fibrin hydrogels crosslinked by thrombin are an excellent platform for exploring effects on DCs owing to thrombin’s biocompatibility and biodegradability. Considering the importance of the interaction between implanted bone plates and host immune system, as shown in Figure 6a–c, our group developed a thrombin-crosslinked fibrin hydrogel for replicating the interfacial fibrin deposition reaction between implanted bone plates and adjacent tissue. The thrombin-crosslinked fibrin hydrogel was developed for exploring the influences of dimensionality on the shape deformation, cytoskeleton reorganization, and mechanical performance of DCs [13].

### 2.2. Hydrogels with Noncovalent Interactions

Many noncovalent linkages motifs have been reported for hydrogel formation [54], including hydrogen-bond interactions, electrostatic interactions, hydrophobic interactions, and host–guest interactions.

#### 2.2.1. Hydrogen-Bond Interactions

**Figure 7:**
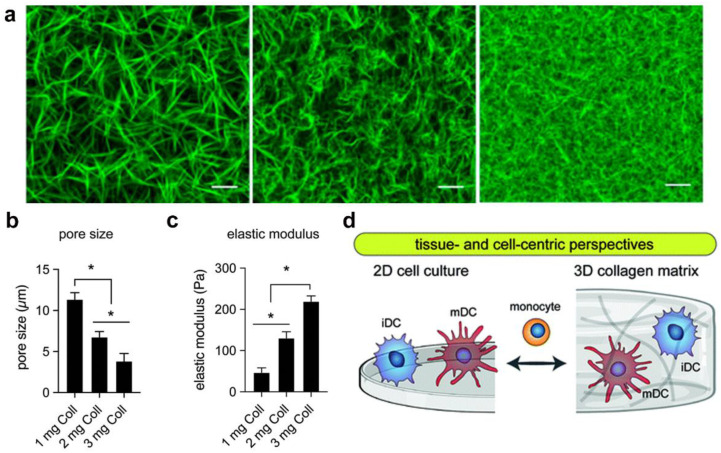
Collagen hydrogel matrices for DC culture. (**a**) Collagen hydrogels with different fibrillar microarchitectures (reprinted/adapted with permission from Ref. [12], 2020, Sapudom et al.) (**b**) Topological and (**c**) mechanical properties of collagen matrices with various concentrations (* significance level of *p* < 0.05). (**d**) Schematic diagram of a collagen hydrogel for DC culturing (Reprinted/adapted with permission from Ref. [60], 2017, Xie et al.).

Hydrogen bonds are weak diatomic interactions that play an important role in natural macromolecular systems, commonly involving a hydrogen atom and electronegative atom, such as oxygen or nitrogen. Specific protein–protein interactions and polysaccharide–polysaccharide interactions are among the most well-studied platforms for cell–matrix interactions owing to their biocompatibility, low immunogenicity, and resemblance to parts of the natural ECM [55], such as collagen and agarose. As shown in Figure 7a, collagen is the most abundant structural protein in the ECM; thus, hydrogels based on collagen are among the most well-studied platforms for cell–matrix interactions [56,57]. Rapid pH changes can drive collagen self-assembly to develop hydrogen bond-crosslinked hydrogels [58]. Additionally, the topological and mechanical properties of collagen networks can be adjusted with the collagen concentration (Figure 7b,c) [12]. The formed collagen hydrogel can provide a powerful platform for DC functions [14,57,59], such as three-dimensional migration and mechanosensory mechanisms (Figure 7d).

**Figure 8:**
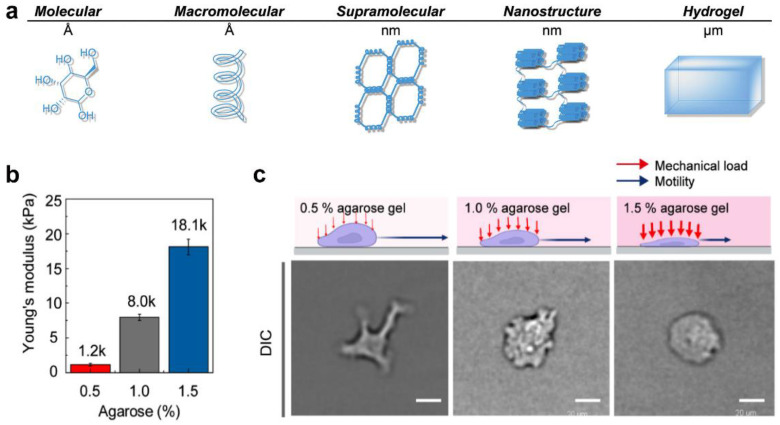
Agarose hydrogel with varied mechanical stiffness for DC culture. (**a**) Agarose hydrogel based on hydrogen-bond interactions. (**b**) The Young’s moduli of agarose hydrogels with different concentrations. (**c**) Agarose hydrogels with various mechanical stiffnesses modulated DCs’ morphology. (Reprinted/adapted with permission from Ref. [63], 2021, Yoon-Kyoung Cho et al.).

Agarose is a neutral thermo-responsive polysaccharide that can dissolve in water with heat treatment and rapidly gelatinize during a temperature drop, which is mainly attributable to the structural change between a random-coil configuration and double helices with multiple chain aggregation [61]. As shown in Figure 8a–c, agarose can be developed as a hydrogel confiner that reveals the effects of mechanical loads on DC migration within a confined microenvironment, in which different stiffnesses are realized by changing the agarose concentrations [11,62,63]. For example, as the agarose concentrations increased from 0.5 *w/v*% to 1.5 *w/v*%, the stiffnesses increased from 1.2 to 18 kPa, which mimicked the wide range of stiffness of tissues in the human body [11]. The formed agarose hydrogel confiner is different from the microfabricated PDMS, as described in the previous section, and DCs fully confined within the agarose hydrogel with different stiffnesses were compelled to adapt their cellular morphology [63].

#### 2.2.2. Electrostatic Interactions

**Figure 9:**
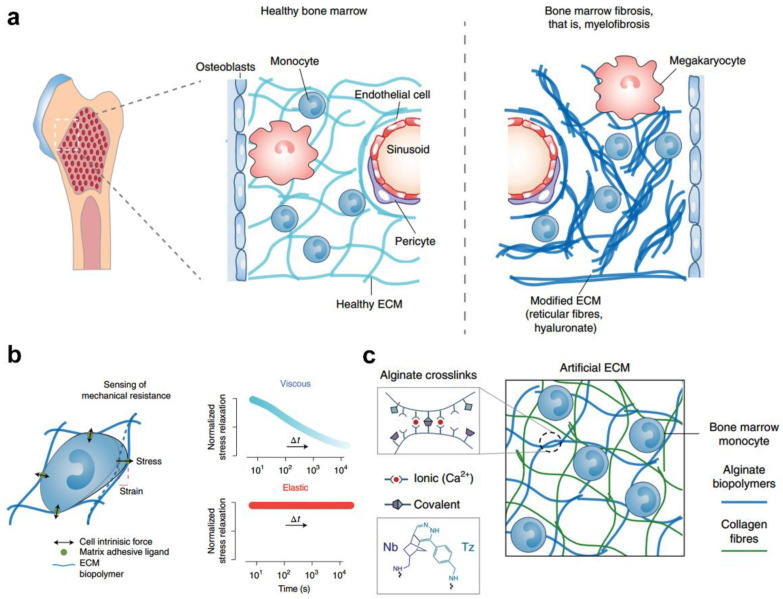
Alginate and collagen interpenetrating network hydrogel for regulating monocyte differentiation in fibrotic niches. (**a**) In myeloproliferative neoplasms, abnormal haematopoiesis is associated with inflammation and substantial ECM remodeling. (**b**) Bone marrow cells sense the stiffness and viscoelasticity of the matrix. (**c**) Alginate was chemically modified with norbornene (Nb) and tetrazine (Tz), which covalently reinforce existing ionic (Ca^2+^) hydrogel crosslinks to modulate viscous performance independently of stiffness. (Reprinted/adapted with permission from Ref. [19], 2022, Vining et al.).

Polyelectrolytes derived from natural or synthetic polymers with fixed opposite charges can form electrostatically crosslinked hydrogels [64]. For example, electrostatic interactions favor the formation of alginate hydrogel systems, in which the alginate is readily bound with a divalent calcium ion. Importantly, weak ionic linkages lead to viscoelastic hydrogels that accurately capture extracellular matrix dynamics [65]. In particular, the viscoelasticity is easily regulated through changing different parameters, such as the density of divalent cations, alginate molecular weight, or alginate concentration [66]. Differentiation of monocytes encapsulated within a dynamic 3D microenvironment has also been reported. For example, Mooney et al. reported an interpenetrating network that was created from alginate and collagen, in which the stiffness of the hydrogel was tuned by cooperative binding of divalent cations. Additionally, permanently crosslinked alginate networks with click-chemistry groups can endow the hydrogels with more elastic behavior without changing the gel nanostructure (Figure 9a–c). Human monocytes encapsulated in static or elastic hydrogels system displayed a proinflammatory polarization phenotype and differentiation toward DCs, which was different from monocytes cultured in viscoelastic hydrogel [19].

#### 2.2.3. Hydrophobic Interactions

The hydrophobic-interaction-based hydrogels that are formed through copolymerizing hydrophobic monomers or a grafting approach usually rely on microphase separation and aggregation of hydrophobic domains. Since the formed hydrogel systems are stable and rarely influenced by ionic strength or pH, the hydrophobic-interaction-based hydrogels might be potential candidates for better engineering of mechanical microenvironments in vitro. Additionally, their mechanical performances can be readily adjusted by changing the density of hydrophobic interactions. As a typical example of hydrophobic-interaction-based hydrogels, temperature-responsive hydrogels can freely flow at low temperatures and transform into stable hydrogels at body temperature [67]. For instance, fibronectin-immobilized temperature-responsive poly(N-isopropylacrylamide) hydrogels were used as a powerful platform for analyzing mechanical signal transduction of encapsulated cells [68].

#### 2.2.4. Host–Guest Interactions

Host–guest supramolecular hydrogels are based on the interaction of one or more guest molecules that are accommodated by a cavitand host, such as cyclodextrins [69,70] and cucurbituril [71]. Bian et al. developed a gelatin host–guest hydrogel that was locally stiffened by surface-modified silica nanoparticles, in which the rigid nanostructures provided local network rigidity and better replicated the dynamic mechanical microenvironment of the native ECM. More importantly, the obtained host–guest supramolecular hydrogels with a heterogeneous network topology facilitated the mechanosensory cues of the encapsulated cells [72].

### 2.3. Hydrogels with Dynamic Covalent Interactions

Hydrogels that associate through reversible covalent interactions can closely replicate the biochemical and mechanical properties of the ECM [25], and thus dynamic covalently crosslinked hydrogels improve the fidelity of cell culture models. Specific examples of hydrogels with dynamic covalent interactions include reversible carbon–nitrogen double-bonds, dynamic covalent boronic esters, and Diels–Alder linkers.

#### 2.3.1. Reversible Carbon–Nitrogen Double-Bonds

**Figure 10:**
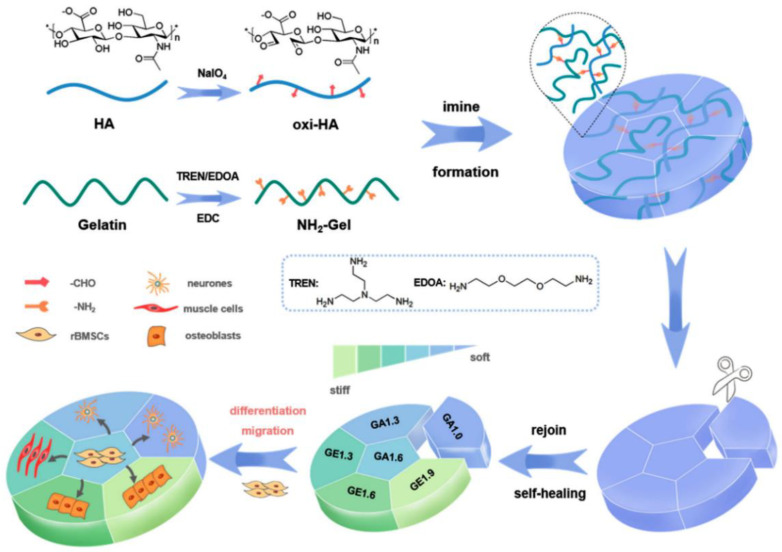
Schematic illustration of the gradient circular-patterned hydrogel for regulating cell behaviors. To build a hydrogel model containing cell-regulating cues, hydrogels with gradient stiffnesses are spliced into a circular pattern based on their self-healing performance. (Reprinted/adapted with permission from Ref. [80], 2022, Wang et al.).

The crosslinking strategies based on condensation reactions between carbonyl groups and nucleophiles are important approaches for developing a dynamic hydrogel platform, in which imine, oxime, or hydrazone linkages occur under physiological conditions [73,74,75]. The reactions are reversible and mild because the only byproduct from the reaction is water; thus, the carbonyl-condensation reactions are different from the traditional covalent linkages, by which, initiators or catalyzers might introduce potential toxicity issues [76]. The Schiff-base reaction refers to the reactions between a primary amine and carbonyl group, resulting in a carbon–nitrogen double-bond, producing an imine bond. Oximes and hydrazones, which structurally resemble imines, can be produced through the condensation reactions of carbonyl groups with α-effect nucleophiles consisting of terminal primary amine groups next to oxygen and nitrogen atoms [77,78]. Due to their reversibility and viscoelasticity, the carbonyl condensation reactions have become an emerging and promising method to prepare hydrogels that better recapitulate dynamic ECM mechanics, which may be beneficial for exploring the effects of ECM mechanics on cellular behaviors or functions [79]. For example, Chen et al. developed a circular-patterned hydrogel platform with gradient stiffness through Schiff-base reactions between oxidized hyaluronic acid and gelatin derivatives (Figure 10). Utilizing their unique self-healable ability resulting from the formation of dynamic covalent bonds, the hydrogels were further spliced into one single specimen, and then the formed hydrogel with gradient stiffnesses ranging from 1.2 to 28.9 kPa was used as an in vitro cell culture platform for studying the influences of stiffness on stem cell adhesion, migration, and differentiation [80].

**Figure 11:**
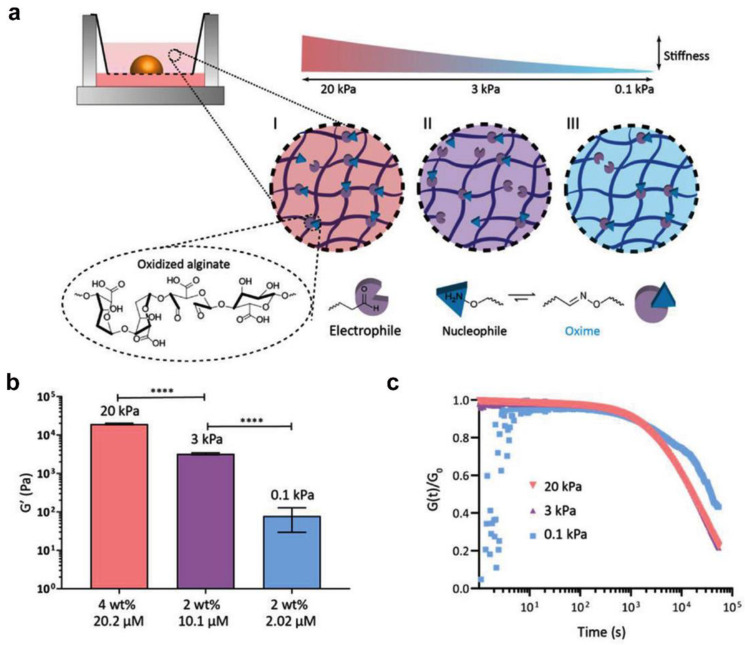
Schematic of the oxime-crosslinked hydrogel system with varying stiffness. (**a**) Schematic diagram of the different hydrogel systems based on oxidized alginate cross-linked with oxime. The I–III plot represents the increase in stiffness of the hydrogel as the cross-linking density increases. (**b**) The shear moduli of different hydrogel compositions (**** significance level of *p* < 0.0001). (**c**) Stress relaxation of the hydrogels. (Reprinted/adapted with permission from Ref. [82], 2022, Ruiter et al.).

Beyond that 2D culture, the carbonyl-condensation reactions can also endow hydrogels with the ability to encapsulate cells [25] or organoids [77]. For example, the matrix adaptability and viscoelasticity displayed in hydrazone-crosslinked (covalent) hydrogels favor the biosynthesis of embedded chondrocytes [65,81]. More recently, Baker et al. reported that the stiffness and stress relaxation of an oxime-crosslinked sodium-alginate hydrogel impacted the renal phenotype and caused undesired fibrotic markers. The oxime-crosslinked hydrogel was developed by mixing the oxidized alginate and a small bifunctional oxime crosslinker, and the stiffness increased from 0.1 to 20 kPa by increasing the concentration of oxidized alginate or oxime crosslinker. The characteristic stress relaxation times changed from 1.6 × 10^4^ to 3.9 × 10^4^ s (Figure 11a–c). A renal organoid encapsulated within a stiff hydrogel displayed signs of an epithelial–mesenchymal transition. Conversely, encapsulation in a stress-relaxing hydrogel platform induced all major renal segments and less fibrosis [82].

#### 2.3.2. Dynamic Covalent Boronic Esters

**Figure 12:**
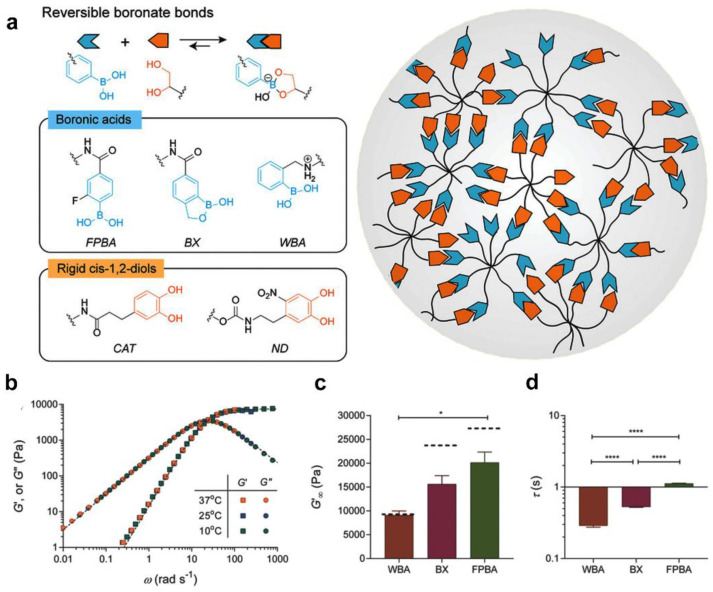
Design of covalent adaptable networks based on dynamic boronate bonds for cell culture. (**a**) Schematic of covalent, adaptable networks based on boronic-ester-crosslinked hydrogels (**b**) Frequency-sweep spectrum of boronate hydrogels. (**c**) Comparisons of the high-frequency plateau moduli (* significance level of *p* < 0.05) and (**d**) the network relaxation times of different hydrogel (**** significance level of *p* < 0.0001). (Reprinted/adapted with permission from Ref. [87], 2018, Tang et al.).

Boronic-ester-crosslinked hydrogels have emerged as an effective approach for exploring cell–matrix interactions, in which a cyclic ester is formed through the reversible condensation reaction between diols and boronic acids under physiological conditions and is free from any catalyst [83]. Particularly, different static or dynamic mechanical properties can also be achieved by using simple neighboring group effects [84,85]. Therefore, boronic-ester-crosslinked hydrogels have become a promising approach to developing a fast relaxation hydrogel platform for studying cellular mechano-transduction. Additionally, the reversibility of boronic-ester-crosslinked hydrogels allow for 3D cell encapsulation [86]. Anseth et al. developed boronic-ester-crosslinked hydrogels by rationally selecting boronic acid variants and diols, and a tunable viscoelastic rheological performance was achieved by changing the structure of the boronic acid (Figure 12a–d). In in vitro 3D cell culture models, the fast relaxation matrix mechanics favored cell–matrix interactions [87]. In addition to tissue regeneration, viscoelasticity also plays an important role in fibrotic disease progression. A boronic-ester-crosslinked hydrogel system with the highest levels of stress relaxation activated valvular interstitial cells, in which the characteristic myofibroblast markers, such as collagen 1a1 and α-smooth muscle actin, were prominently upregulated [88].

#### 2.3.3. Diels–Alder Reaction

**Figure 13:**
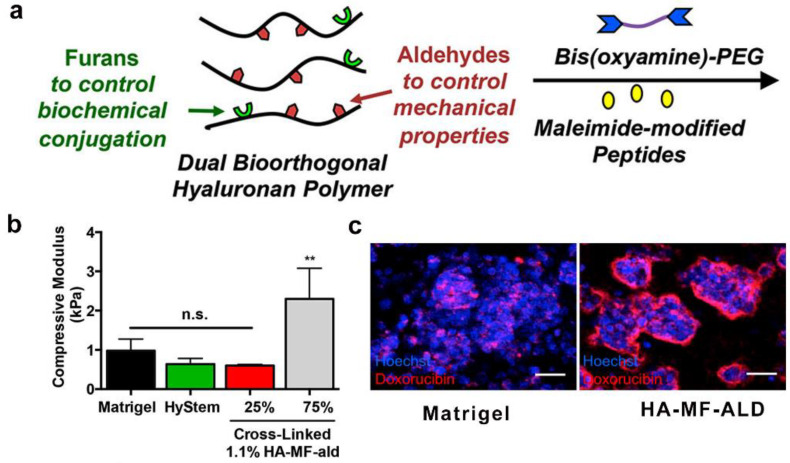
Hyaluronan-based hydrogels with oxime and Diels−Alder chemistry to culture breast cancer spheroids. (**a**) Schematic of preparation of hyaluronic-acid-based hydrogels. (**b**) Tunable mechanical performance of hyaluronic acid-based hydrogels (** significance level of *p* < 0.01, n.s. = not significant). (**c**) Hyaluronic-acid-based hydrogels for culturing breast cancer spheroids (Reprinted/adapted with permission from Ref. [93], 2017, Baker et al.).

A Diels–Alder reaction is a [4 + 2] cycloaddition reaction in which a cyclohexene adduct is formed between conjugated diene and a substituted alkyne or alkene [89,90]. Consequently, Diels–Alder linkers have been used to develop in vitro cell culture platforms [91,92]. For example, Shoichet et al. developed a hyaluronic acid (HA)-based hydrogel with an independently tunable stiffness and peptide composition for recapitulating the tumor microenvironment [93], in which the mechanical and biochemical performances were adjusted by oxime ligation and Diels–Alder chemistry, respectively. Aldehyde-group-modified HA was first synthesized, and then the methyl furan motifs were subsequently immobilized. Oxime ligation can be formed between the HA-aldehyde groups and bis(oxyamine)-PEG, whereas maleimide-functionalized peptides can react with HA-methylfuran groups based on a Diels–Alder reaction. The optimized hydrogel facilitated the formation of breast cancer spheroids that epitomized drug-resistant tumors (Figure 13a–c).

## 3. Engineering Hydrogels Control DC Function

**Figure 14:**
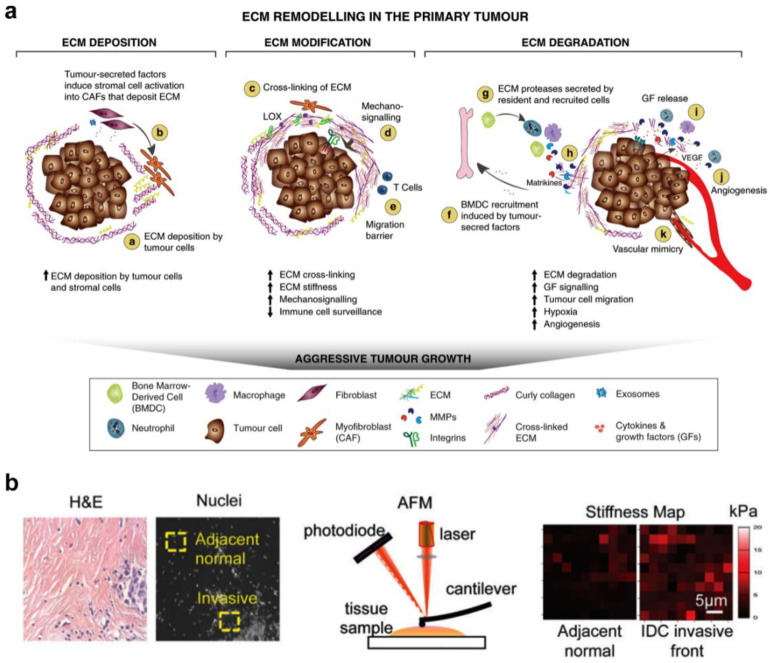
ECM remodeling in a primary tumor. (**a**) The matrix stiffness results from crosslinking of the ECM in tumor progression (reprinted/adapted with permission from Ref. [97], 2020, Winkler et al.). (**b**) The distribution of stiffness between the tumor invasion front and adjacent healthy tissue (reprinted/adapted with permission from Ref. [98], 2015, Acerbi et al.).

DCs work as sentinel cells in the immune system, patrolling the blood and peripheral tissue to detect numerous danger signals, such as foreign antigens or pathogens. In surveying tissues, DCs travel long distances through the body, encountering different biophysical cues throughout their life cycle, such as stiffnesses and topology [94]. The stiffness of most soft tissues ranges between 2 and 5 kPa. Conversely, muscles and bones display higher physiological stiffness ranging from 10 to 20 GPa [95,96]. The stiffness of healthy tissues can change under pathological conditions. Accumulating evidence has demonstrated that immune-related pathological conditions such as fibrosis and tumors are associated with tissue stiffening [97]. Tumor initiation and progression generate complex structural changes in the ECM, which can lead to differences in local ECM stiffness, a feature that distinguishes them from normal physiological stiffness (Figure 14a). For instance, healthy breast tissues display a physiological stiffness of about 400 Pa, whereas fibrotic breast tissues can reach a stiffness upwards of 20 kPa in rare cases (Figure 14b) [98,99,100]. Another typical example is lymph nodes that stiffen in an inflammatory microenvironment, which correlates directly with abnormal ECM remodeling [101]. Thus, biophysical cues generated from different ECM mechanical and topographical characteristics might affect DC function. Given the emerging importance of biophysical cues for DC behavior, it is essential to understand their impact on DC function in both physiological and pathological contexts. Engineering hydrogels with tunable mechanical performance or topology holds great promise for matching the different ECM microenvironments. In this section, recent advances in developing hydrogel platforms with tunable mechanical performance or topology for controlling DC function are highlighted (Table 1), including the morphology, migration, immunophenotype, and immune functions of the DCs.

### 3.1. DC Morphology

**Figure 15:**
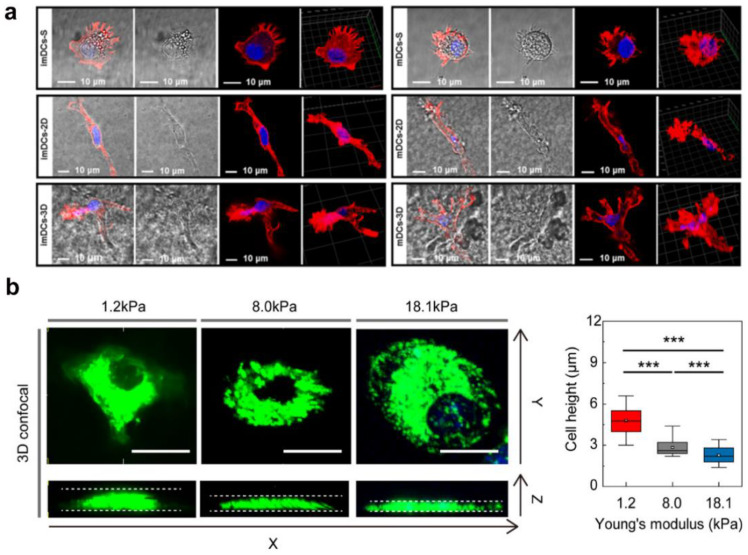
Matrix dimensionality and stiffness regulate DC morphology. (**a**) Representative confocal images of the cytoskeletons of DCs cultured on 2D and in 3D fibrin hydrogels (reprinted/adapted with permission from Ref. [13], 2022, Hu et al.). (**b**) Morphological changes in DCs cultured in confining agarose hydrogels with different stiffnesses (*** significance level of *p* < 0.005) (reprinted/adapted with permission from Ref. [11], 2021, Choi et al.).

DCs were originally identified in 1973 by a Canadian scientist named Steinman, and eventually became known as “dendritic cells” owing to the distinctive stellate-like cytoplasmic protrusive morphological features during the maturation stages [104]. The morphological changes in DC, such as the formation of veils and dendrites, are closely associated with migration, motility, phagocytosis, and DC–T cell contacts. DC morphological changes induced by biochemical factors were reported in our previous studies, and mainly included tumor-derived cytokines [6], such as IL-10 [9], TGF-β1 [7,8], and VEGF [10]. In addition to biochemical cues, changes in DC morphology may depend on biophysical cues, such as dimensionality and stiffness. As shown in Figure 15a, F-actin is mainly located at the extreme ends of DCs and away from the nuclei in a suspension culture. Interestingly, DCs cultured on a 2D fibrin matrix display an elongated morphology, resulting in a predominantly bi-polarized shape, whereas DCs encapsulated in a 3D fibrin matrix spread in several directions, resulting in a predominantly tri-polarized or stellate spindle-shaped morphology [13]. The distinctive changes in DC morphology are related to RhoA and CDC42 levels. In addition to the culture dimensionality, substrate stiffness also modulates DC morphology. For example, adaptive responses of DCs cultured in agarose gels with different compressive force levels were clearly visible as morphological differences. As shown in Figure 15b, the height of DCs was distinctively increased at a low mechanical load (1.2 kPa), whereas DCs were more flattened with increased mechanical loads ranging from 8.0 to 18.1 kPa. Consequently, the collected results highlighted that dimensionality and stiffness modulated DC morphology [11].

### 3.2. DC Migration

**Figure 16:**
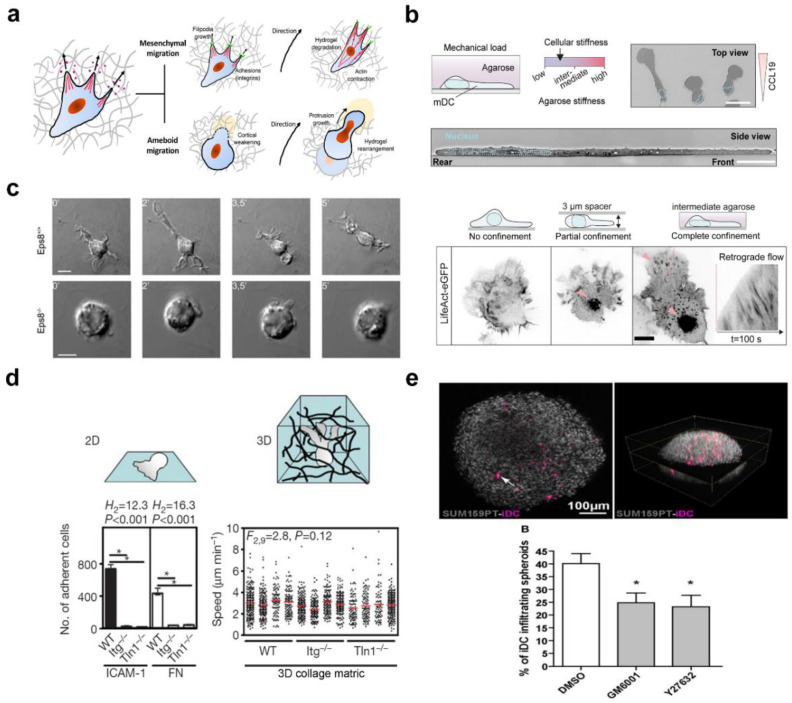
DC migration mode with different dimensionalities and stiffnesses. (**a**) Schematic of mesenchymal migration and amoeboid migration (reprinted/adapted with permission from Ref. [109], 2021, Franck et al.). (**b**) Confinement of DCs under agarose with different stiffnesses, in which the actin patches form in response to confinement (reprinted/adapted with permission from Ref. [63], 2022, Gaertner et al.). (**c**) Esp8-/- BMDCs fail to extend elongated cell protrusions and migrate in 3D collagen hydrogels. (Reprinted/adapted with permission from Ref. [103], 2011, Emanuela et al.). (**d**) Migration of DCs cultured in 2D and 3D collagen hydrogels (reprinted/adapted with permission from Ref. [14], 2008, Lämmermann et al.). (**e**) DCs adopt both amoeboid and mesenchymal migration in tumor spheroids (* significance level of *p* < 0.05) (reprinted/adapted with permission from Ref. [110], 2018, Cougoule et al.).

As the sentinels of the immune system, DCs are a heterogeneous population that reside in both lymphoid organs and peripheral tissues, in which DCs experience distinctive tissue microenvironments with a wide range of stiffnesses. Most importantly, effective immune responses depend on optimized cell migration with trafficking of distinct DC subsets across peripheral or lymphoid tissues [105,106]. Therefore, it is very important to understand how biophysical cues modulate the intrinsic motility of DCs [107]. As shown in Figure 16a, there are two distinct migration patterns that have been identified for DCs, ameboid-like migration and mesenchymal migration [108]. The ameboid-like migration is independent of cell adhesivity and usually requires porous environments without proteolytic degradation or junction interactions. For example, Michael et al. utilized DCs as a model system to understand how ameboid cells responded to microenvironments with different degrees of confinement [63]. As shown in Figure 16b,c, the migratory behaviors of DCs were explored by using agarose hydrogels with different stiffnesses. DCs displayed small actin-rich patches embedded in a homogeneous actin cortex when migrating in an intermediate stiffness agarose hydrogel, and the formed patches were scattered across the entire cell; there were peaks in intensity in the cell bodies. The underlying molecular mechanism is based on the Wiskott–Aldrich syndrome proteins that assemble into dot-like structures, providing activation platforms for Arp2/3 nucleated actin patches. These patches locally push against the external load, which can be obstructing collagen fibers or other cells, and thereby create space to facilitate forward locomotion. In 2011, Giorgio et al. reported that DCs encapsulated in a collagen hydrogel required the actin capping activity of the signaling adaptor Eps8 to polarize, resulting in elongated migratory protrusions [103]. As shown in Figure 16d, Michael et al. reported that DC migration was integrin-dependent on 2D substrates, whereas migration of DCs encapsulated in a 3D collagen hydrogel was independent of integrin [14]. The distinctive DC migration in the 3D microenvironment was mainly attributable solely to the force formed by the actin network’s expansion, which promoted protrusive flow of the leading edge. Subsequently, myosin II-dependent contraction promoted contraction of the trailing edge that propelled the rigid nucleus, resulting in a purely protrusive mode of migration. Unlike ameboid-like migration, mesenchymal migration that is traction-dependent is closely related to strong adhesive interactions with the surrounding matrix, proteolytic degradation, and ECM remodeling [109]. In 2018, Isabelle et al. revealed that DCs adopted two migration modes in 3D collagen hydrogel [110]. As shown in Figure 16e, amoeboid migration was observed in a porous hydrogel matrix, whereas DCs adopted the ROCK-independent and protease-dependent mesenchymal migration mode in a dense hydrogel network.

**Figure 17:**
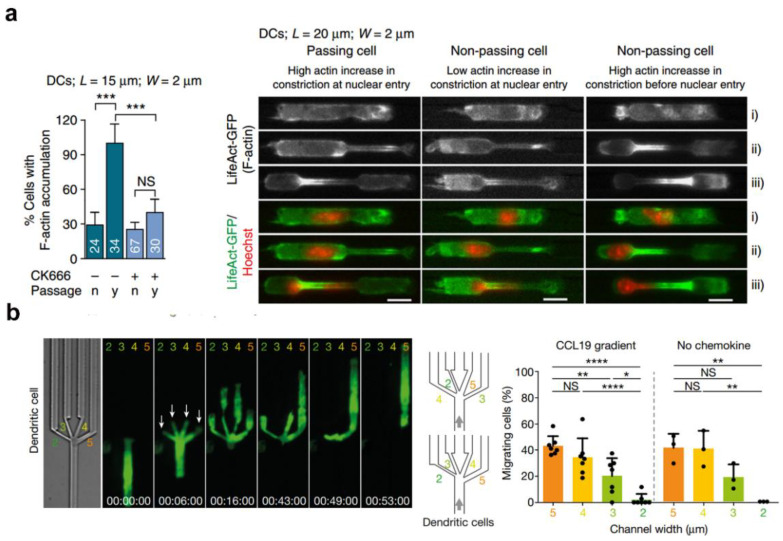
DC migration in narrow spaces. (**a**) Actin polymerization enables nuclear deformation to facilitate cell migration through complex environments (*** *p* < 0.001 as determined by one-way ANOVA with Tukey’s test) (reprinted/adapted with permission from Ref. [15], 2016, Thiam et al.). (**b**) Protrusion dynamics of the leading edge of a DC, during migration in microchannels through a junction (decision point) with four differently sized pores (one-way ANOVA with Tukey’s test; **** *p* < 0.0001, ** *p* = 0.0029, * *p* = 0.0225) (reprinted/adapted with permission from Ref. [111], 2019, Renkawitz et al.).

Cell migration has two opposite effects, which are advantageous immune responses under physiological processes and detrimental effects resulting from infiltration of cancer cells. In vivo, DC migration occurs in heterogeneous microenvironments, calling for high cellular deformability that is usually restricted by the cell nucleus. In 2016, Matthieu et al. reported an underlying migration mechanism of DCs based on a micrometric constrictions model [15]. As shown in Figure 17a, DCs displayed strong enrichment of actin filaments around a nucleus inside a small constriction, indicating that the enrichment was temporally restricted to the time during which the nucleus was deformed in the constriction and spatially limited to the constriction. The migration mechanism of DCs is closely associated with rapid Arp2/3-dependent actin nucleation around the nucleus, which disrupts the nuclear lamina, the main structure limiting nuclear deformability [15]. In 2019, to further understand how DCs navigated through dense tissues, as shown in Figure 17b, Matthieu et al. revealed that DCs confined in large pores chose the path of least resistance [111], which allowed them to circumnavigate local obstacles and effectively follow global directional cues, such as chemotactic gradients. Distinctive frontward positioning of the nucleus was observed with changes in the size of the pore, which enabled DCs to use their bulkiest compartment as a mechanical gauge. Once the nucleus and the closely associated microtubule organizing center passed the through largest pore, the cytoplasmic protrusions still lingering in smaller pores were retracted.

### 3.3. DC Phenotype and Differentiation

DCs develop through a complex differentiation and maturation process that is usually accompanied by a change in phenotype and in function. Monocyte-derived DCs display unique phenotypic and functional characteristics that distinguish them from all other APCs. Generally, monocytes first differentiate into immature dendritic cells (imDCs) that display a high endocytic capacity. Subsequently, imDCs patrol the microenvironment of peripheral tissues to detect antigens. Upon encountering antigens, imDCs internalize the antigens, and then they become mature DCs (mDCs) during their maturation stage, resulting in upregulated expression of the major histocompatibility complex (MHC) and accessory molecules (such as CD11c, CD80, CD83, CD86, and CCR7) on their surfaces. Under a high concentration of CCL21 in draining lymph nodes (LNs), mDCs move to LNs via afferent lymph vessels and present antigens to T cells, resulting in the initiation of acquired immunity. In addition to biochemical cues, increasing evidence has demonstrated that DC phenotype and differentiation can be regulated by the biophysical microenvironment. Engineered hydrogels are a powerful platform for studying the phenotype and differentiation of DCs owing to the ability to recapitulate diverse biochemical microenvironments, including the microenvironment’s stiffness [17,18,31], dimensionality [12,13,59], and pore structure [16].

**Figure 18:**
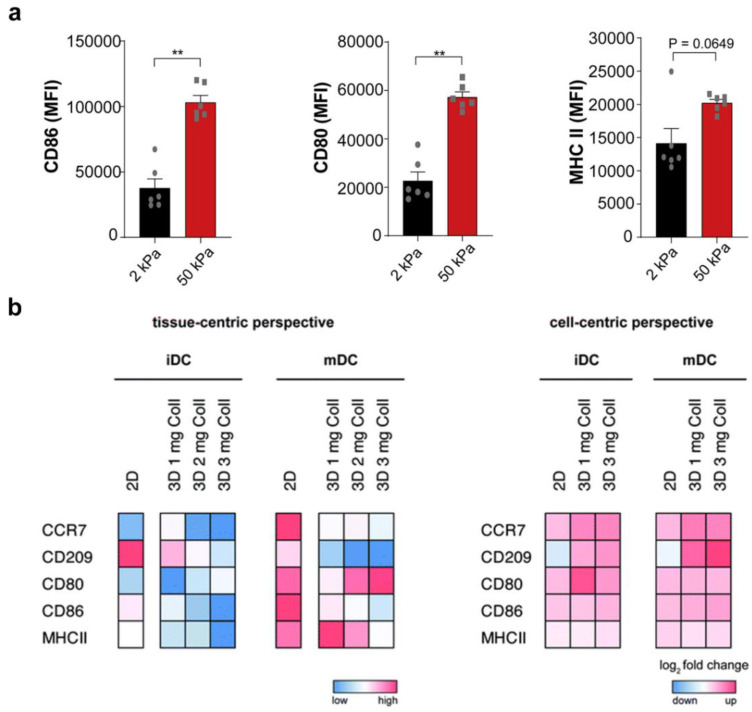
Dimensionality and stiffness modulate DC phenotype. (**a**) Surface expression of CD80, CD86, and MHC class II from BMDCs on PDMS substrate in which black dots and white squares correspond to the different test values that BMDCs on 2 kPa and 50 kPa, respectively. (* *p* < 0.05, ** *p* < 0.01, *** *p* < 0.001 as determined by Mann-Whitney U test) (reprinted/adapted with permission from Ref. [17], 2021, Chakraborty et al.). (**b**) DC immune phenotype on 2D and 3D collagen hydrogels (reprinted/adapted with permission from Ref. [12], 2020, Sapudom et al.).

Tissue stiffness is defined as the capacity of resistance to deformation. Tissue stiffness is well known to change in an immune-related pathological context, such as tumor or fibrosis progression. It has been confirmed that DC phenotype and differentiation are affected by substrate stiffness. For example, a PAM hydrogel with tunable substrate stiffness upregulated the CD83 expression on mDCs and CD86 expression on imDCs [18]. As shown in Figure 18a, BMDCs cultured on a 50 kPa PDMS hydrogel substrate mimicking fibro-inflammatory disease displayed high levels of CD80, CD86, and MHCII in response to stimulation with LPS [17]. Conversely, BMDCs cultured on a 2 kPa hydrogel substrate representing physiological resting stiffness displayed reduced activation and cytokine production. Commonly, BMDCs cultured on plastic dishes are susceptible to becoming activated during cell culturing, which is unfavorable for imitating the internal immune function. To solve this dilemma, Xiang et al. reported a novel photo-crosslinked hydrogel substrate for culturing BMDCs. BMDCs cultured in the optimized gelatin hydrogel substrate with 5 kPa stiffness displayed a phenotype and function similar to those of spleen DCs in vivo and were capable of facilitating T cell stimulation after LPS activation [31]. In addition, it was reported that DCs cultured within a 3D hydrogel network were more similar to their in vivo counterparts in comparison with those cultured on 2D hydrogel substrates [12,13,59]. As shown in Figure 18b, Teo et al. reported that cell culture dimensionality modulated the differentiation of DCs. From a tissue-centric perspective, surface marker expression (such as CCR7, CD209, CD80, CD86, and MHC II) of both imDCs and mDCs cultured on a 2D substrate was generally higher in comparison with those cultured in a 3D collagen hydrogel. Conversely, from a cell-centric perspective, upregulated expression of cell surface markers in both imDCs and mDCs was found. In 2017, Jing et al. explored whether 3D collagen hydrogel favored BMDCs differentiating into specialized DCs. They found that BMDCs encapsulated in a 3D microenvironment differentiated into a distinct subset of DCs, displaying low expression of CD11c, CD40, CD80, CD83, CD86, and MHC-II molecules in comparison with those cultured on a 2D substrate [59]. Overall, these findings identify substrate stiffness and dimensionality as critical biophysical cues impacting the activation and maturation of DCs, which is of great importance in the treatment of many clinical diseases, such as tumor immunity and infection-induced inflammation.

### 3.4. DC Immune Functions

**Figure 19:**
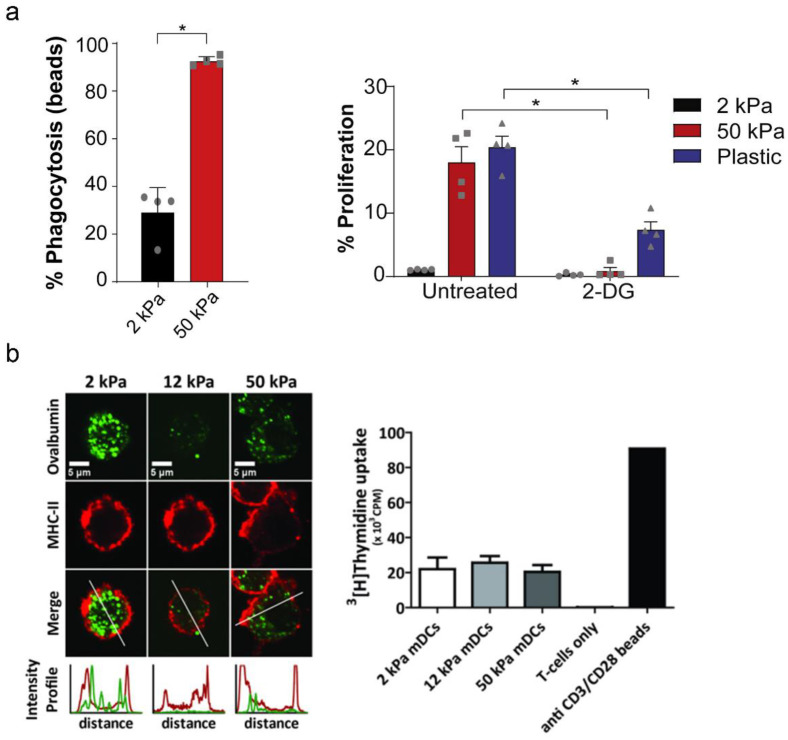
Mechanical stiffness controls DC immune functions, such as (a and b) phagocytosis and T cell proliferation. (**a**) BMDCs cultured on PDMS hydrogel with different stiffness. (**b**) Human peripheral blood monocyte- derived DCs cultured on PAM hydrogel with different stiffness in which gray dots, squares and triangles correspond to the different test values that BMDCs on 2 kPa, 50 kPa and plastic, respectively. (* *p* < 0.05, ** *p* < 0.01, *** *p* < 0.001 as determined by Mann-Whitney U test.) (reprinted/adapted with permission from Ref. [17], 2021, Chakraborty et al.; Reprinted/adapted with permission from Ref. [18], 2017, Mennens et al.).

As a specialized APC, DCs play a central role in bridging innate and acquired immunity. DCs first express plentiful phagocytic receptors for efficiently phagocytizing pathogens. Endocytosed proteins are internalized by DCs and then degraded by lysosomal proteolysis, resulting in the formation of peptides [112,113]. MHC molecules bind with peptides, and then the formed peptide-loaded MHC molecules are delivered to the cell surface in the process of DC maturation. Finally, the activated DCs transmit antigen signals to naive T cells, resulting in T cell differentiation into different types of Th cells [114]. The activation and maturation of DCs are coupled with rapid and sustained glycolytic reprogramming. Upon TLR ligation, BMDCs rapidly upregulate the glycolytic flux to meet their bioenergetic and biosynthetic demands. In 2021, Winer et al. found that DC metabolism was independent of TLR agonist input and was closely associated with matrix stiffness. Higher stiffness upregulated glucose metabolism in DCs to support their inflammatory phenotype in comparison with those cultured on a pliant hydrogel, which was related to the major Hippo-signaling factor, TAZ, and Ca^2+^-related ion channels, including potentially Piezo1, as tension sensors in DCs. As shown in Figure 19a, BMDCs cultured to a stiffness of 50 kPa displayed an increased capacity for phagocytosis. Additionally, an enhanced antigen-presenting capacity of BMDCs was observed in plastic, or 50 kPa [17]. Similarly, Liu et al. reported that DCs cultured on a stiffened PDMS hydrogel substrate upregulated the expression of Piezo1, which altered the secretion of inflammatory factors, such as TGFβ1 and IL-12, that directed the reciprocal differentiation of TH1 and regulatory T cells [102]. Conversely, as shown in Figure 19b, the antigen endocytosis of DCs displayed opposite stiffness-dependent differences, and a DC culture on PAM substrates of 2 kPa took up 1.5–2-fold more ovalbumin in comparison with those cultured at 12 and 50 kPa [18].

## 4. Conclusions and Perspectives

In the past few decades, multidisciplinary approaches have contributed remarkable advances in the development of engineered hydrogels to understand how the biophysical microenvironment affects DC function. Although DC mechanobiology has been broadly explored in 2D hydrogel microenvironments, DC mechanobiology in 3D hydrogel microenvironments is still deficient and controversial due to the complex crosstalk between biochemical and biophysical signaling pathways. As such, it is very important to precisely decouple biophysics from biochemistry for future research. Additionally, a better understanding of the potential molecular mechanisms underlying DCs’ adaptive migration ability that allows them to effectively execute their effector functions in response to different microenvironment is needed. This may answer the question of how DCs carry out their individual immune functions in a physiological or pathological context.

Recent advances in the design of a hydrogel platform with tunable biophysical performance (stiffness, dimensionality, and topological structure, etc.) have offered a versatile toolbox for modulating DC function in vitro [115,116]. Having reviewed the latest progress in engineering hydrogels for modulation of DC function, we herein draw conclusions on the challenges and potential directions for the future. Firstly, despite the versatile modulation of the biophysical performance of hydrogels, they are still limited in recapitulating the spatiotemporal physical cues in vivo resulting from the complexity and heterogeneity of the native mechanical microenvironments of DCs. Current functional dynamic hydrogels that can precisely capture ECM dynamics may provide potential hydrogel candidates for better engineering hydrogels for modulation of DC function. Additionally, the development of cancer-on-a-chip systems may also make considerable contributions to engineering hydrogels for modulation of DC function. Secondly, the mechanisms of how three-dimensional spatiotemporal biophysical cues affect DC function remain under debate because of the crosstalk and interplay between signaling pathways and microenvironmental cues. As such, it is necessary to precisely characterize DC responses (e.g., morphology, migration, and phenotype) to spatiotemporal biophysical cues. Finally, DCs cultured in enzymatically or chemically crosslinked hydrogel platform usually need enzymatic or chemical hydrogel digestion to collect cells, which may affect DC function. The temperature-responsive hydrogels may provide potential candidates for the release of DC in the absence of enzyme.

## Figures and Tables

**Table 1:** Summary of hydrogel types used for modulating the DC functions.

Hydrogel	Gelation Mechanisms	Reinforcing Mechanism	DC Type	Cellular Response	Ref.
PDMS	Covalent interactions	Covalent bond interactions	BMDCs	BMDCs cultured on plastic dishes are susceptible to becoming activated during cell culture. DCs cultured on a stiffened PDMS hydrogel substrate upregulated the expression of Piezo1, that directed the reciprocal differentiation of TH1 and regulatory T cells.	[17,102]
PAMs	Covalent interactions	Covalent bond interactions	Human peripheral blood monocyte- derived DCs	A PAM hydrogel with tunable substrate stiffness upregulated the CD83 expression on mDCs and CD86 expression on imDCs. DCs culture on PAM substrates of 2 kPa took up 1.5–2-fold more ovalbumin in comparison with those cultured on 12 and 50 kPa.	[18]
Photo-crosslinked polymer	Covalent interactions	Covalent bond interactions	BMDCs	BMDCs cultured in the optimized gelatin hydrogel substrate displayed parallel phenotypes and functions relative to spleen DCs in vivo and were capable of facilitating T cell stimulation after lipopolysaccharide (LPS) activation.	[31]
Fibrin	Covalent interactions	Covalent bond interactions	BMDCs	DCs cultured on a 2D fibrin matrix display an elongated morphology, whereas DCs encapsulated in a 3D fibrin matrix spread in several directions.	[13]
Collagen	Noncovalent interactions	Hydrogen bonds interactions	BMDCs	DCs encapsulated in a collagen hydrogel required the actin capping activity of the signaling adaptor Eps8 to polarize, resulting in elongated migratory protrusions. DC migration was integrin-independent in a 3D collagen hydrogel.	[12,14,103]
Agarose	Noncovalent interactions	Hydrogen bonds interactions	BMDCs	DCs cultured in agarose gels with different compressive force were clearly visible as morphological differences. DCs displayed small actin-rich patches embedded in a homogeneous actin cortex when migrating in an intermediate stiffness agarose hydrogel, and the formed patches were scattered across the entire cell, with peak intensities in the cell body.	[11,63]
Alginate	Noncovalent interactions	Electrostatic interactions	Human monocytes	Human monocytes encapsulated in static or elastic hydrogels system displayed a proinflammatory polarization phenotype and differentiation toward DCs.	[19]
